# Ciprofloxacin is a potential topoisomerase II inhibitor for the treatment of NSCLC

**DOI:** 10.3892/ijo.2012.1653

**Published:** 2012-10-04

**Authors:** TOMASZ KLOSKOWSKI, NATALIA GURTOWSKA, JOANNA OLKOWSKA, JAKUB MARCIN NOWAK, JAN ADAMOWICZ, JAKUB TWORKIEWICZ, ROBERT DĘBSKI, ALINA GRZANKA, TOMASZ DREWA

**Affiliations:** 1 Departments of Tissue Engineering; 2 Histology and Embryology, Nicolaus Copernicus University, Bydgoszcz;; 3 Laboratory of Clinical and Experimental Oncology, Department of Pediatric, Hematology and Oncology Nicolaus Copernicus University, Bydgoszcz;; 4 Department of Urology, Institute of Oncology, Kielce, Poland

**Keywords:** lung cancer, ciprofloxacin, cytostatic effect, F-actin

## Abstract

Lung cancer is one of the most common tumors and its treatment is still inefficient. In our previous work we proved that ciprofloxacin has a different influence on five cancer cell lines. Here, we aimed to compare the biological effect of ciprofloxacin on cell lines representing different responses after treatment, thus A549 was chosen as a sensitive model, C6 and B16 as highly resistant. Three different cell lines were analyzed (A549, B16 and C6). The characterization of continuous cell growth was analyzed with the Real-Time Cell Analyzer (RTCA)-DP system. Cytoskeletal changes were demonstrated using immunofluorescence. The cell cycle was analyzed using flow cytometry. Ciprofloxacin was cytostatic only against the A549 cell line. In the case of other tested cell lines a cytostatic effect was not observed. Cytoskeletal analysis confirms the results obtained with RTCA-DP. A549 cells were inhibited in the G2/M phase suggesting a mechanism related to topoisomerase II inhibition. The biological effects of ciprofloxacin support the hypothesis that this drug can serve as an adjuvant treatment for lung cancer, due to its properties enabling topoisomerase II inhibition.

## Introduction

Lung cancer is one of the most common tumors, every year over one million people die from this cancer (1). Currently, the management of non-small cell lung cancer is ineffective and insufficient, which is why it is necessary to search for new drugs that will replace or assist those currently used (2).

Ciprofloxacin is an antibiotic that belongs to the fluoroquinolone class (3). This group of antibiotics has a broad spectrum (4,5). Ciprofloxacin also inhibits topoisomerase II in eukaryotes, including mammalian cells (6,7).

Previously we showed that ciprofloxacin can act against lung cancer cells using trypan blue and MTT assays (8). These methods give little information on this drug and its influence on lung cancer cells *in vitro*(8–11). In the current study, we aimed to present cell viability in a long-term continuous culture system with analysis of the subcellular architecture and the cell cycle. To show the possible difference the highly resistant lines B16 and C6 were chosen for the experiments.

## Materials and methods

### Cell lines

A549 (human non-small cell lung cancer) and C6 (rat glioblastoma) cell lines were purchased by the American Type Culture Collection (Manassas, VA). The B16 (mouse melanoma) cell line was established from B16 tumors excised from C57BL/6J mouse. A549, B16 and C6 cell lines were cultured in DMEM/HAM’S F-12 medium containing 10% of fetal bovine serum (FBS), supplemented with 5 *μ*g/ml amphotericin B, 100 *μ*g/ml streptomycin and 100 U/ml penicillin. All cell lines were grown in 25-cm^2^ Nunc T-flasks at 36°C and 5% CO_2_.

### Lethal Concentration calculation

Lethal concentration (LC) values were calculated using trypan blue assay. The results are presented in Table I(8).

### Proliferation assay

**Figure 1:**
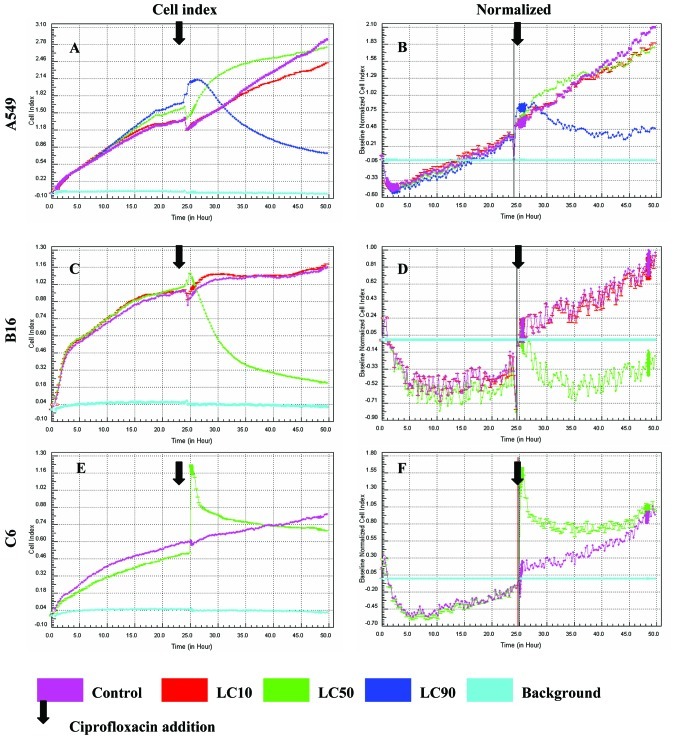
Proliferation assay. Cell index and normalization results obtained after 24-h incubation with ciprofloxacin on RTCA-DP analyzer.

**Figure 2:**
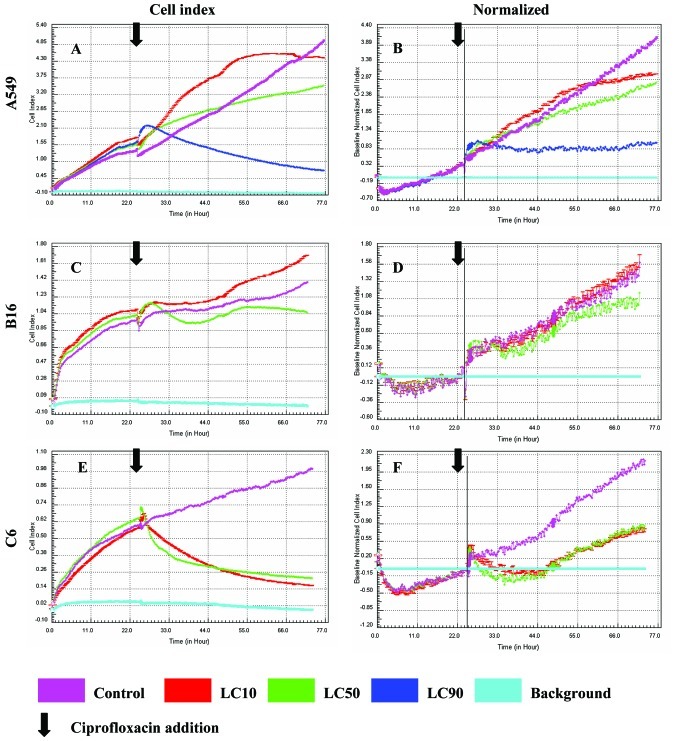
Proliferation assay. Cell index and normalization results obtained after 48-h incubation with ciprofloxacin on RTCA-DP analyzer.

The influence of ciprofloxacin on different cancer cell lines was established using the Real-Time Cell Analyzer (RTCA)-DP that belongs to the xCELLigence system (Roche Applied Science). This enables monitoring cellular events in real time without the incorporation of levels. Cells (n=6250) were seeded on each well of E-Plate 16 (Roche Applied Science). Cells were incubated in normal cell culture medium for 24 h. In the next step ciprofloxacin in concentrations corresponding to LC values (Table I) was added. LC values for ciprofloxacin were previously established (8). Cells were incubated with ciprofloxacin for 24 and 48 h. The viability of cells has been expressed by means of cell index (CI). Cell index values are the results of the increase in electrode impedance on E-Plate by the increase in cell number attached to the electrodes. Cell index values can be used to monitor the viability, number, morphology and adhesion degree in a number of cell-based assays. A medium without a cell culture served as the background. The results are presented in two ways: as an effect of cell index and after normalization of these results. Normalization allows reference of the obtained results of ciprofloxacin cytotoxicity to the control and the background. For each well, the normalized cell index is calculated as the cell index at a given point divided by the cell index at the normalization time point. Thus, the normalized cell index for all wells must equal 1 at the normalization time point (for example when a tested compound was added). Due to normalization we can determine the mode of action of the tested drug, as cytotoxic or cytostatic. The experiments were carried out in triplicate. Figure curves are presented as mean values of three experiments (Figs. 1 and 2).

### Fluorescent staining of F-actin

Cells were seeded on cover slips in 24-well plates (density, 1×10^4^ cells/well). After 24 h of incubation cells were treated with ciprofloxacin for 48 h in doses according to LC values (Table I). Then cells were washed in PBS and fixed in paraformaldehyde (15 min, 37°C). After washing in PBS (3×5 min), cells were incubated with 0.1 Triton X-100 (Serva Electrophoresis, Heidelberg, Germany) in HBSS (5 min), washed in PBS (3×5 min) and labeled with phalloidine conjugated with Alexa 488 [dilution 1:40, 20 min, room temperature (RT), darkroom] (Invitrogen Life Technologies, Carlsbad, CA). Cover slips were washed 3×5 min in PBS before counterstaining for DNA with DAPI (100 ng/ml, 10 min, RT, darkroom) (Sigma). Finally cells were washed in distilled water before mounting in Aqua-Poly/Mount (Polysciences, Inc.) Stained cells were analyzed using NIS-Elements 4.0 software in Eclipse E800 fluorescence microscope (Nikon).

### Cell cycle analysis

Cells were seeded on 24-well plates at a density of 5×10^4^ cells/well and exposed to ciprofloxacin at concentrations corresponding to LC values (Table I). After 24 h of incubation cells were washed twice with PBS solution, detached from wells with trypsin and then centrifuged for 5 min at 300 x g at 4°C. In the next stage 500 *μ*l of hypotonic, DNA coloring solution of PI/Triton X-100 were added. The solution consisted of 50 *μ*g/ml PI, 0.1 mg/ml RNAase and 0.05% Triton X-100. Cells were incubated for 30 min in the dark at room temperature. Then, in order to stop the reaction, the tubes with reaction mixture were placed on ice and then transferred to a flow cytometer equipped with System IITM Software, Version 1.0. (Coulter Electronics, Krefeld, Germany). The results obtained after 24 h in LC50 for C6 cells were not analyzed in cytometer because of high ciprofloxacin concentration and small number of cells obtained after incubation. The experiments were carried out in triplicate.

## Results

### Proliferation assay

Proliferation assays are presented in Figs. 1 and 2, after 24 and 48 h respectively. Ciprofloxacin was most effective against the A549 lung cancer cell line. We observed a decrease in viability at concentrations corresponding to LC10, LC50 and LC90. The graphs obtained after cell index normalization showed that ciprofloxacin acted as a cytostatic but not cytotoxic compound in the case of A549 cells after 24 and 48 h in LC90 (Figs. 1B and 2B). In the concentration corresponding to LC10 and LC50 a cytostatic effect on A549 cells was not observed (Figs. 1B and 2B).

No significant decrease of the viability of B16 cells was observed except for LC50 after 24 h, but after normalization a cytostatic effect was not achieved (Fig. 1C). A cytostatic effect was not achieved either after 24 and 48 h for all concentrations in the case of the C6 glioblastoma cell line after result normalization (Figs. 1F and 2F).

### Fluorescent staining of F-actin

**Figure 3:**
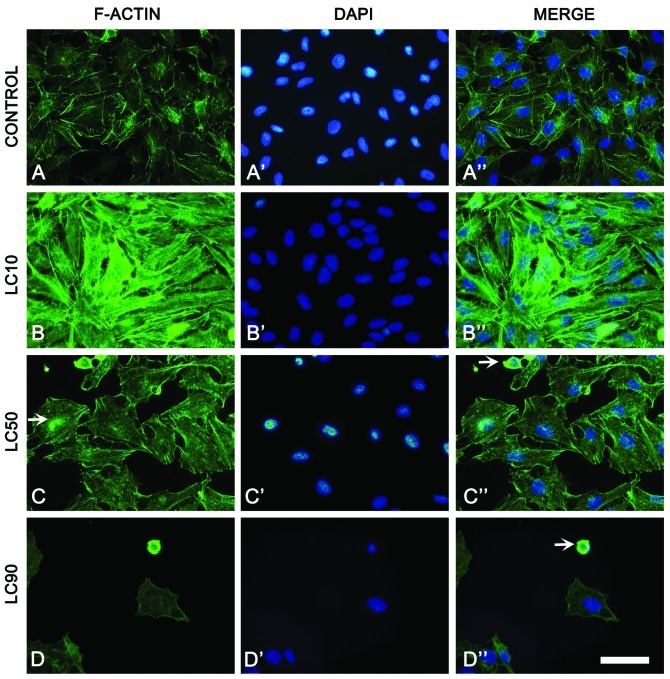
Cytoskeletal visualization of the A549 cell line using fluorescent microscopy. (C) Arrows mark cell shrinkage and (C″, D″) rounded cells which condensed actin. Bar, 100 *μ*m.

Changes in cytoskeletal organization were observed in non-small cell lung cancer cells treated with ciprofloxacin. LC10 caused increase of F-actin fluorescence. However, ciprofloxacin treatment (LC50, LC90) induced changes of F-actin distribution (decreased number of actin fibers), leading to cell shrinkage. In LC50, actin aggregates were observed in the cytoplasm. Furthermore, rounded cells with condensed actin in LC50 and LC90 were present. In the highest dose of ciprofloxacin no actin fibers were observed in A549 cells (Fig. 3). A few apoptotic cells with cell blebbing were also observed in highest concentration of ciprofoxacin (data not shown).

**Figure 4:**
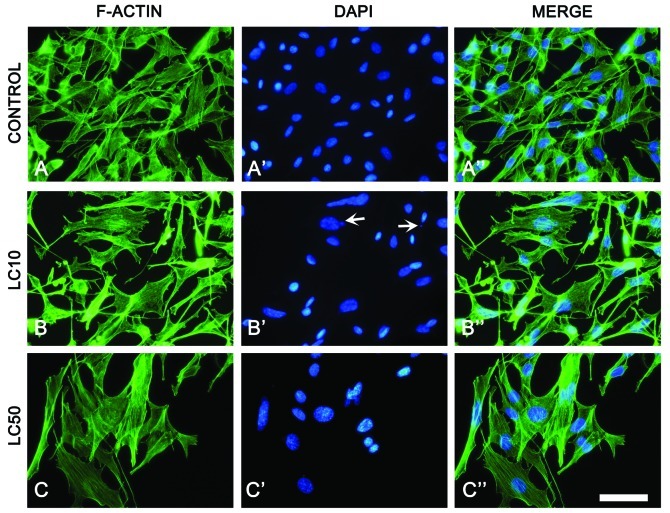
Cytoskeletal visualization of the B16 cell line using fluorescent microscopy. (B′) Arrows mark apoptotic bleb formation. Bar, 100 *μ*m.

The B16 cell line treated with ciprofoxacin showed more extensive cytoskeletal organization that resulted in cell enlargement especially in LC50. DAPI staining revealed alternation in size (increase) and shape of the nucleus after incubation with the drug, but apoptotic bleb formation was also observed (Fig. 4).

**Figure 5:**
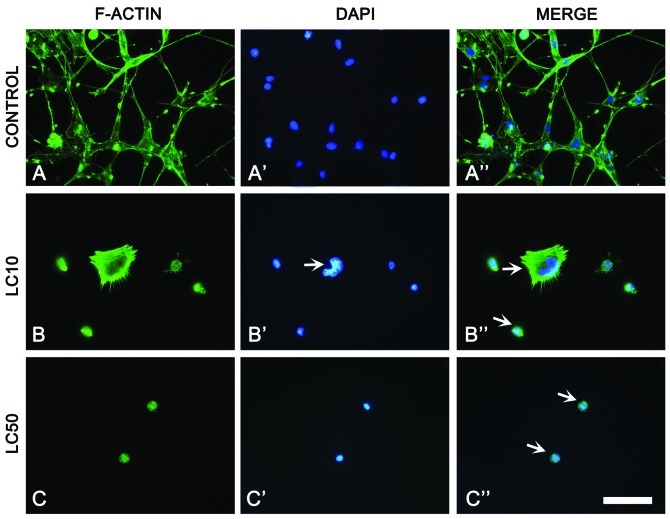
Cytoskeletal visualization of the C6 cell line using fluorescent microscopy. (B″, C″) Arrows mark shrunken and rounded cells with condensed actin and (B′, B″) larger cells with irregular nuclei. Bar, 100 *μ*m.

Fluorescent microscopy showed elongated shape and some intercellular junction in the C6 cell line. Actin fibers were visible. Ciprofloxacin treatment caused loss of intracellular connection and cell shrinkage (LC10, LC50). Shrunken and rounded cells with condensed actin were observed. However larger cells with irregular nuclei shape were also observed (Fig. 5).

### Cell cycle analysis

**Figure 6:**
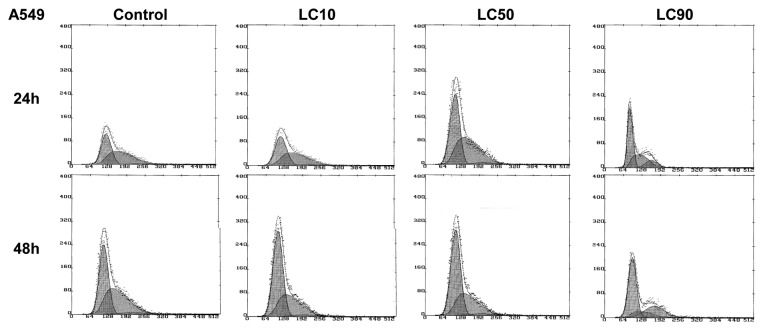
Cell cycle analysis. Results of cell cycle analysis obtained for the A549 cell line.

The A549 cell cycle control study showed that after a short incubation time (24 h), a large number of cells was in the S phase, even up to half of the population. This reflected a high prolifaration rate at the beginning. This number decreased with the incubation time, which was associated with the proliferation rate inhibition after 48 h as a result of contact inhibition. This effect was also observed for LC10 and LC50. In the highest concentration (LC90) G2/M accumulation was observed, which indicated the arrest of the cell cycle in this phase (Fig. 6).

**Figure 7:**
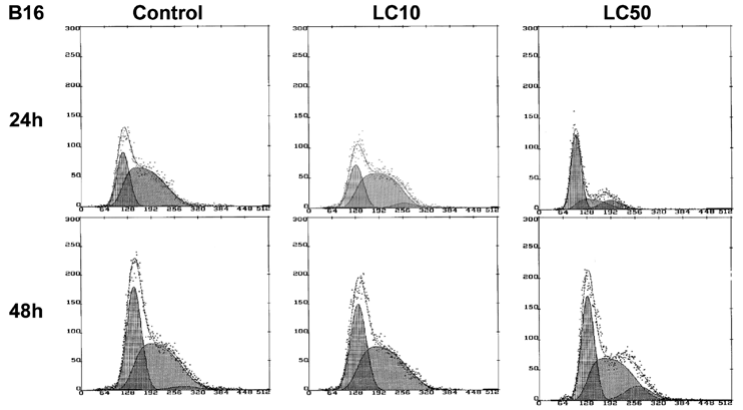
Cell cycle analysis. Results of cell cycle analysis obtained for the B16 cell line.

In case of the B16 cell line, the LC90 value could not be established. In the control group, after a short incubation time (24 h) melanoma cells proliferated intensively (67% of cells in S phase), after a longer incubation time (48 h) up to 52% of cells were in the S phase. Similar results were observed in LC10, indicating that this concentration does not affect B16 cells. In LC50, a higher amount of cells in the G2/M phase was observed indicating G2/M block after 48 h (Fig. 7).

**Figure 8:**
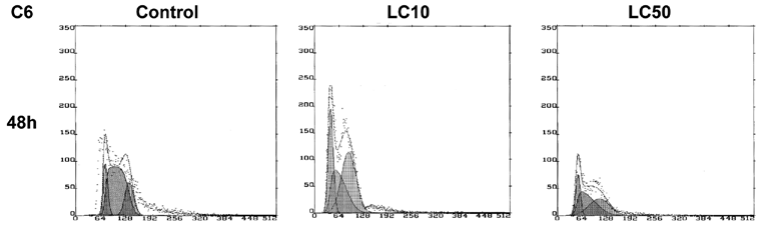
Cell cycle analysis. Results of cell cycle analysis obtained for the C6 cell line.

The results of the cell cycle analysis for C6 glioblastoma cells were significantly different from two previously described cell lines. In control, cells still proliferated intensively after 48 h. With the increasing ciprofloxacin concentration we observed a decrease in the number of cells in the S phase, (from 57-49% in LC50) with predominance of G1/G0 and G2/M phases. The cell cycle was not established for 24 h due to impossible to established values of LC10 and LC90 (Table I) (8). The LC50 concentration led to intensive proliferation and unpredictable behavior of the cell cycle of C6 cells incubated with ciprofloxacin (Figs. 1E and 8).

## Discussion

xCELLigence system is a relatively new method. This system measured cell viability in real time. Apoptosis occurs only during a short period of time, often within hours, that is why continuous viability monitoring is a useful tool that can show how the examined drug acts against cells. This system is able to distinguish the cytotoxic effect from the inhibition of proliferation. The continuous monitoring of cell viability by the xCELLigence system makes it possible to distinguish different perturbations of cell viability, such as senescence, cell toxicity (cell death) and reduced proliferation (cell cycle arrest) (12). Thus, this system can help to predict biological *in vivo* effect exerted by the tested drug.

Ciprofloxacin exerted cytostatic effect against A549 cells (lung cancer), but not against B16 (melanoma) and C6 cells (glioblastoma). The most effective action of ciprofloxacin was directed against lung cancer cells. Only in the case of the A549 cell line, all LC values (10, 50, 90) could be calculated and examined based on our previous work (8). The cell index based on the experiment performed on the xCELLigence system indicates better toxic effectiveness in relation to A549 cells than B16 and C6 cells. A significant decrease in the viability in concentration corresponding to LC50 after a 48 h incubation was observed for A549 cells (Fig. 2A). Results obtained after F-actin staining confirm the results obtained by the xCELLigence system. We observed a decrease in the cell index (xCELLigence) (Fig. 2A) and the cell number (fluorescence microscopy) (Fig. 3D) with increasing concentration of drug. DAPI staining showed that one of the possible A549 cell death mechanisms is apoptosis, single cells with apoptotic blebs were visible in LC90. Most cells had very condensed F-actin and chromatin, cells without stress fibers were also observed (Fig. 3). The results obtained from cell cycle analysis showed that ciprofloxacin influenced the inhibition of proliferation and the accumulation of cells in G2/M phase (Fig. 6). G2/M accumulation can potentially lead to the induction of cell death after ciprofloxacin treatment, but it has to be proven. The supposed mechanism of action leads to G2/M accumulation is a topoisomerase II inhibition (13,14). This means that ciprofloxacin can be a potential topoisomerase II inhibitor for A549 cells.

In the case of the B16 cell line, LC90 could not be established. Lack of cytostatic effect on B16 cells induced by ciprofloxacin was recorded in LC50. In LC10, the viability of cells was observed to be even higher than in control (Table I, Figs. 1C and 2C). This was probably due to the predominant activation of prosurvival pathways over the apoptotic pathways in lower concentrations of ciprofloxacin (15). This phenomenon was described as hormesis (16,17). This phenomenon was accompanied by changes in F-actin distribution. Results obtained in fluorescence microscopy indicate that B16 cells reduced in number and increased in size with increasing ciprofloxacin concentration (Fig. 4). This may explain the lack of cytostatic effect after a 48 h incubation, because this system measures the level of well surface coverage and not the cell number. DAPI staining showed nuclei blebs which can indicate that ciprofloxacin can induce apoptosis in the B16 cell line (Fig. 4B′). G2/M accumulation was also observed after 48 h in LC50 (Fig. 7), confirming that ciprofloxacin inhibited topoisomerase II (13). Thus, the mode of action is topoisomerase II inhibition.

The weakest action of ciprofloxacin was observed in the case of the rat glioblastoma cell line. In this case, the LC90 value was impossible to establish. The calculated LC values were the highest compared to the other tested cell lines, A549 and B16. No cytostatic effect was observed after ciprofloxacin addition. After F-actin staining, cell shrinkage and F-actin condensation was observed. Cells lost their long cytoplasmatic lammelopodia which reduced their attachement to the well surface (Fig. 5). These results are the confirmation of the cell viability test (Fig. 2E). The changes in the cell cycle after incubation with ciprofloxacin were unspecific (Fig. 8). All the above observations lead to a conclusion that C6 cells were highly resistant to ciprofloxacin treatment.

Aranha *et al*(18) analyzed the influence of ciprofloxacin on the cell cycle against the HTB9 bladder cancer cell line. They showed that after a 72- and 96 h incubation with this drug at a concentration of 300 *μ*g/ml, cells accumulated in the S and G2/M phase (10% more cells in the S phase and 28–34% in the G2/M phase as compared to control). We have shown that a shorter incubation time could exert a cytostatic effect in G2/M accumulation in A549 lung cancer cells.

The influence of this antibiotic on a different NSCLC line, NCI-H460, was examined by Mondal *et al*(19). They showed that a lethal concentration value (LC50) after a 48 h ciprofloxacin treatment was as low as 19.5 *μ*g/ml (in our study 102.1 *μ*g/ml), which confirmed the effectiveness of ciprofloxacin action against lung cancer.

Chemotherapeutic agents can act in two ways: inhibit cell growth (cytostatic) or kill cells (cytotoxic) (7). Toxic ciprofloxacin action against cancer cells depends on topoisomerase II inhibition. We also observed G2/M inhibition and showed its properties as a cytostatic agent.

The anticancer effect against lung cancer can be enhanced due to the ability of ciprofloxacin to accumulate in higher concentrations in lung tissue than in serum after an intravenous (i.v.) and oral administration. The excellent penetration of ciprofloxacin from the blood into the lung parenchyma was confirmed soon after an oral drug delivering. The maximum oral dosage of ciprofloxacin prescribed to patients is 750 mg twice a day. Drug dose after an oral administration reaches 4.3 *μ*g/ml concentration in the serum. The concentration of ciprofloxacin after an oral administration is up to 7 times higher in the lung tissue than in the serum (20). This indicates that the maximum concentration of ciprofloxacin after an oral dose of one 750 mg tablet can reach 30.1 *μ*g/ml. After a long-term therapy this concentration can be even higher. In some cases a patient can take 1 g oral dose of ciprofloxacin twice a day for 20 months (21). Ciprofloxacin penetrates into the lung parenchyma also after an intravenous administration. After a high-dose treatment (800 mg every 12 h for 8 days, i.v.) ciprofloxacin can reach a serum concentration of 13 *μ*g/ml (potential concentration in the lung 91 *μ*g/ml) (22). Such a high-dose of the drug did not cause any side effects and do not cause the induction of bacterial resistance. The minimal inhibitory concentration of ciprofloxacin for most bacteria is 2 *μ*g/g (5). After an administration of 300 mg i.v. ciprofloxacin can reach a concentration of 7.3 *μ*g/g. Ciprofloxacin can be administered intrapulmonarily. Administration of 200 *μ*g/kg of ciprofloxacin can reach 40 and 20 *μ*g/ml values in alveolar macrophages and epithelial lining fluid, respectively. This method allows administration of lower doses of ciprofloxacin compared to the oral route (23). LC50 after 48 h (100 *μ*g/ml) can be theoretically achieved *in vivo* in lung tissue (Table I). This indicates that ciprofloxacin can be added to conventional NSCLC chemotherapy as an adjuvant drug with a different mechanism of action.

Ciprofloxacin seemed to be a good candidate for experimental therapy for lung cancer and an antibiotic of choice for the treatment of lung cancer with concomitant inflammation.

## Figures and Tables

**Table I:** LC values for cell lines: A549, B16 and C6.

		LC values (*μ*g/ml)		
Cell line	Time of incubation with ciprofloxacin (h)	LC10	LC50	LC90
A549	24	27.7	133.3	593.2
	48	18.2	102.1	389.5
B16	24	23.8	409.5	NA
	48	12.0	81.0	NA
C6	24	NA	5915.0	NA
	48	572.0	1092.0	NA

NA, not assessed.
